# Characterization of Ribeye Subunits in Zebrafish Hair Cells Reveals That Exogenous Ribeye B-Domain and CtBP1 Localize to the Basal Ends of Synaptic Ribbons

**DOI:** 10.1371/journal.pone.0107256

**Published:** 2014-09-10

**Authors:** Lavinia Sheets, Matthew W. Hagen, Teresa Nicolson

**Affiliations:** 1 Department of Otology and Laryngology, Harvard Medical School, Boston, Massachusetts, United States of America; 2 Eaton-Peabody Laboratory, Massachusetts Eye and Ear Infirmary, Boston, Massachusetts, United States of America; 3 Oregon Hearing Research Center and Vollum Institute, Oregon Health & Science University, Portland, Oregon, United States of America; Texas A&M University, United States of America

## Abstract

Synaptic ribbons are presynaptic structures formed by the self-association of RIBEYE–the main structural component of ribbon synapses. RIBEYE consists of two domains: a unique N-terminal A-domain and a C-terminal B-domain that is identical to the transcription co-repressor C-terminal binding protein 2 (CtBP2). Previous studies in cell lines have shown that RIBEYE A-domain alone is sufficient to form ribbon-like aggregates and that both A- and B- domains form homo-and heterotypic interactions. As these interactions are likely the basis for synaptic-ribbon assembly and structural plasticity, we wanted to examine how zebrafish Ribeye A- and B- domains interact with synaptic ribbons *in vivo*. To that end, we characterized the localization of exogenously expressed Ribeye A- and B- domains and the closely related protein, CtBP1, in the hair cells of transgenic zebrafish larvae. Unexpectedly, exogenously expressed Ribeye A-domain showed variable patterns of localization in hair cells; one zebrafish paralog of A-domain failed to self-associate or localize to synaptic ribbons, while the other self-assembled but sometimes failed to localize to synaptic ribbons. By contrast, Ribeye B-domain/CtBP2 was robustly localized to synaptic ribbons. Moreover, both exogenously expressed B-domain/CtBP2 and CtBP1 were preferentially localized to the basal end of ribbons adjacent to the postsynaptic density. Overexpression of B-domain/CtBP2 also appeared to affect synaptic-ribbon composition; endogenous levels of ribbon-localized Ribeye were significantly reduced as hair cells matured in B-domain/CtBP2 transgenic larvae compared to wild-type. These results reveal how exogenously expressed Ribeye domains interact with synaptic ribbons, and suggest a potential organization of elements within the ribbon body.

## Introduction

Sensory receptors of the visual, auditory, and vestibular systems contain presynaptic specializations that enable them to transmit sensory information with high fidelity and for extended periods of time [1], [2]. These specializations, called dense bodies or synaptic ribbons, are electron-dense structures that tether numerous glutamate-filled vesicles and provide a scaffold for active zone proteins [3], [4]. Synaptic ribbons are thought to support the rapid response of sensory receptors to stimuli by clustering presynaptic calcium channels [5], [6] and retaining a large, rapidly releasable pool of synaptic vesicles at the active zone [7]. They also play a critical role in responding to sustained stimuli by both priming synaptic vesicles for release [8] and replenishing active sites with vesicles for continuous release [9].

Sensory receptors in different organs and species have distinct coding requirements, and synaptic ribbon architecture likely contributes to synaptic function–synaptic ribbons are found in a variety of different sizes and shapes, from generally spherical in hair-cell synapses [10], [11] to plate-like structures in photoreceptor synapses [3]. Moreover, synaptic ribbon structures are dynamic; synaptic ribbons in the retina and pineal gland change their shape in response to illumination [12], [13] or diurnal cycle [14], and this structural plasticity is thought to optimize ribbon synapse function for the corresponding sensory organs in dark versus light conditions [15]. Since variations in synaptic-ribbon size and shape appear to play an important role in optimizing the functional properties of the synapse, different sensory receptors likely use unique molecular mechanisms to establish and maintain their ideal synaptic-ribbon structure. Yet one common mechanism of synaptic-ribbon assembly and growth employed by all sensory receptors is self-association of the unique ribbon synapse protein RIBEYE [5], [16], [17].

RIBEYE is the main structural component of synaptic ribbons [18], [19]. It consists of two domains: an N-terminal proline-rich A-domain and a C-terminal B-domain that is identical to C-terminal binding protein 2 s (CtBP2s)–a splice isoform of the transcriptional corepressor CtBP2 that lacks a nuclear localization signal and may play an important role in regulating intracellular membrane dynamics [20]. Individual RIBEYE subunits form both homo- and heterotypic interactions between three binding sites in their A-domain and two binding sites in their B-domain [17]. Molecular regulation of the homo- and heterotypic interactions between RIBEYE A-domains and B-domains likely controls the assembly and plasticity of synaptic ribbons [21]. Previous studies examining heterologous expression of individual RIBEYE domains have observed that the A-domain alone forms ribbon-like punctate aggregates in cell culture, supporting a role for the A-domain as an aggregation domain [17], [18]. By contrast, heterologously expressed B-domain appears diffusely distributed throughout the cells unless co-expressed with the A-domain or full length RIBEYE. Moreover, interactions between the A-domain and B-domain have been shown to be inhibited by low concentrations of NAD(H), suggesting inhibition of heterotypic interactions between A- and B-domains as a potential mechanism of regulating synaptic ribbon assembly [17]. Yet the behavior of heterologously expressed RIBEYE domains in cell culture may not reflect the native behavior of individual RIBEYE domains within intact sensory cells. Examining the localization of RIBEYE A- and B-domains *in situ*; that is, within an intracellular milieu containing all the necessary components to generate synaptic ribbons, should give important further insight into how RIBEYE self-association is regulated.

Here we characterized the intracellular localization of stably expressed exogenous Ribeye A-domain, B-domain, and the closely related protein CtBP1 within hair cells of transgenic zebrafish larvae at 3 and 5 days-post-fertilization (dpf). Our results indicate that Ribeye A-domain alone does not efficiently localize to synaptic ribbons and suggest a potential substructural organization of CtBPs within synaptic ribbons.

## Materials and Methods

### Ethics Statement

This study was performed with the approval of the Oregon Health and Science University Institutional Animal Care and Use Committee as well as the Massachusetts Eye and Ear Infirmary Animal Care Committee, and in accordance with NIH guidelines for use of zebrafish.

### Fish Strains

Transgenic lines were created and maintained in Tübingen, Tupfel Long Fin, and WIK wild-type backgrounds. Adult zebrafish strains were maintained as previously described [22].

### Generation of transgenic lines

Plasmid construction was performed using the Tol 2/Gateway kit (Kwan, 2007). The hair cell specific *myosin6b* promoter was previously cloned into the 5′entry vector, p5E, 228 (Kindt, 2012). Ribeye domains were PCR amplified from cDNA containing full-length *ribeye a* (NCBI Accession Number NM_001195491.1) or *ribeye b* (NM_001015064.1), and *ctbp1* was amplified from a cDNA clone ID: 7995426 (Open Biosystems; NCBI Accession Number BC045280.1). The following primers were used to PCR amplify clones for insertion into the Tol2 Middle-entry vector (pME, 237): *ribeye a* (full length) [5′ggggacaagtttgtacaaaaaagcaggctatgttgatctccagtaagcag3′] and [5′ggggaccactttgtacaagaaagctgggtgggtatacattttgtcttgcaggcc3′]; *ribeye a (A domain)* [5′ggggacaagtttgtacaaaaaagcaggctatgttgatctccagtaagcag3′] and [5′ggggaccactttgtacaagaaagctgggtgacttgtgtctggtgatgc3′]; *ribeye b (A domain)* [5′ggggacaagtttgtacaaaaaagcaggctatgatggcagtgcgag3′] and [5′ggggaccactttgtacaagaaagctgggtgacttgtttccggtgaagc3′]; *ribeye a (B-domain/ctbp2s)* [5′ggggacaagtttgtacaaaaaagcaggctgccaccatgataaggcctcagatcatgaat3′] and [5′ggggaccactttgtacaagaaagctgggtgggtatacattttgtcttgcaggcc3′]; *ctbp1* [5′ggggacaagtttgtacaaaaaagcaggctgccaccatggctctgatggacaaac3′] and [5′ggggaccactttgtacaagaaagctgggtg ttggtcggaagggatgtctc3′]. PCR products were confirmed by restriction digest and gel electrophoresis. Middle-entry (pMe) vectors were generated by BP reaction per the Tol2 Kit (Invitrogen Live Technologies). BP reaction products were confirmed by sequencing with M16 forward and reverse primers. Injection plasmids were created by using LR clonase enzyme (Invitrogen), and then subsequently confirmed by multiple restriction digests and gel electrophoresis. To generate transgenic fish, plasmid DNA and tol2 transposase mRNA were injected into zebrafish embryos as previously described [23].

All nucleotide and protein alignments were performed using Lasergene software (DNAStar).

### Antibodies

We used previously described [5], [11] custom-generated antibodies against *Danio rerio* peptide sequences of the A-domains of Ribeye a (rabbit polyclonal; 1∶250) and Ribeye b (mouse monoclonal IgG2a; 1∶10,000), and the N-terminal region of Vglut3 (rabbit polyclonal; 1∶1000). We also used clone 9E10 monoclonal and rabbit polyclonal purified antibodies (Sigma-Aldrich) to label c-Myc and K28/86 purified antibody (mouse monoclonal IgG1; 1∶500; NeuroMab, Davis, CA) to label MAGUK.

### Immunohistochemistry

Immunohistochemistry was performed as previously described [24] with slight modifications of the fixation times. Zebrafish larvae were fixed with 4% paraformaldehyde/4% sucrose in phosphate buffer with 0.2 mM CaCl_2_ for 4 h (3 dpf) or 6 h (5 dpf) at 4°C. Following rinse, larvae were permeabilized with ice cold acetone and blocked with phosphate buffered saline (PBS) containing 2% goat serum, 1% bovine serum albumin (BSA), and 1% dimethyl sulfoxide (DMSO). Then they were incubated with primary antibodies diluted in PBS buffer containing 1% BSA and 1% DMSO overnight at 4°C, followed by diluted secondary antibodies coupled to Alexa 488, Alexa 647 (Molecular Probes, Invitrogen), or *DyLight 549* (Jackson ImmunoResearch).

### Confocal Imaging

Z-stack images of whole neuromasts (spaced by 0.5 µm over 7–10 µm) were acquired as previously described [24] with either a Zeiss LSM 700 laser scanning confocal microscope with a 63×1.4 NA Plan-Apochromat oil-immersion objective or a Leica TCS SP5 confocal microscope with a PL APO 63×1.3NA glycerol-immersion objective. For each experiment, the microscope parameters were adjusted using the brightest wild-type specimen such that just a few pixels reached saturation in order to achieve the greatest dynamic range in our experiments.

### Image processing

Digital images were processed using ImageJ software. 3D isosurface renderings were created using Amira 3D Analysis software (FEI Visualization Sciences Group). Subsequent image processing for display within figures was performed using Photoshop and Illustrator software (Adobe).

### Image analysis

Quantitative image analysis was performed on raw images using Amira 3D Analysis software. To reduce background noise, a Gaussian smoothing convolution was applied to each x-y image within a stack with a kernel size of 3 and a σ value of 0.4. To quantitatively measure immunolabel intensity, a user-defined inclusive threshold was applied to isolate pixels occupied by immunolabeled Ribeye spheres or MAGUK patches. The inclusive threshold values for each label were determined using 3D isosurface renderings, with the minimum threshold value defined as the value above which the user could resolve two closely adjacent spheres or patches. Minimum threshold values were typically 80 (of 256) for Ribeye and 110 (out of 256) for MAGUK. The Material Statistics function was then used to measure the cumulative intensity of fluorescent pixels (sum of the grayscale values) within each individual sphere or patch. Ribbon-synapse localized Ribeye spheres were identified as such by juxtaposing MAGUK patches.

### RT-PCR and qPCR

At 5 dpf, groups of 30 transgenic larvae or WT siblings were anesthetized on ice and decapitated with fine surgical scissors to separate the posterior lateral line hair cells of the tail from other ribbon synapse containing tissue in the head. Larval tail tissue was immediately placed into RNAlater (Applied Biosystems/Ambion) and total RNA was extracted using the RNAqueous 4-PCR kit (Applied Biosystems/Ambion). Reverse transcription (RT)-PCR was performed the using 5 ug total RNA and the Sprint RT Complete Oligo(dT) kit (Clontech). For qPCR, 0.2 ul cDNA in SsoFast Supermix (Bio-Rad) with appropriate primers was used for each qPCR reaction, and the reactions were run in 96-well plates using a Bio-Rad CFX96 Real-Time System. The RNA level for *ribeye b* was first calculated from a cDNA standard curve, then normalized to β-actin RNA. Primers used for *ribeye b* transcript are as follows: forward 5′-agttgatgcgcaaaggag-3′ and reverse 5′-atggtggacacgatgactg-3.

## Results

### Zebrafish Ribeye A-domains differ in their self-association and synaptic ribbon localization

The A-domain of RIBEYE contains three specific interaction sites that are thought to mediate RIBEYE self-association [17]. When transfected into HEK293, COS, and R28 cells, the A-domain has been shown to form discrete ribbon-like aggregates [17], [18], suggesting that the A-domain functions primarily as an aggregation domain [18]. We therefore characterized the localization of stably expressed Ribeye A-domain *in situ* by examining its localization in zebrafish stably expressing Ribeye A-domain in lateral-line neuromast (NM) hair cells.

**Figure 1:**
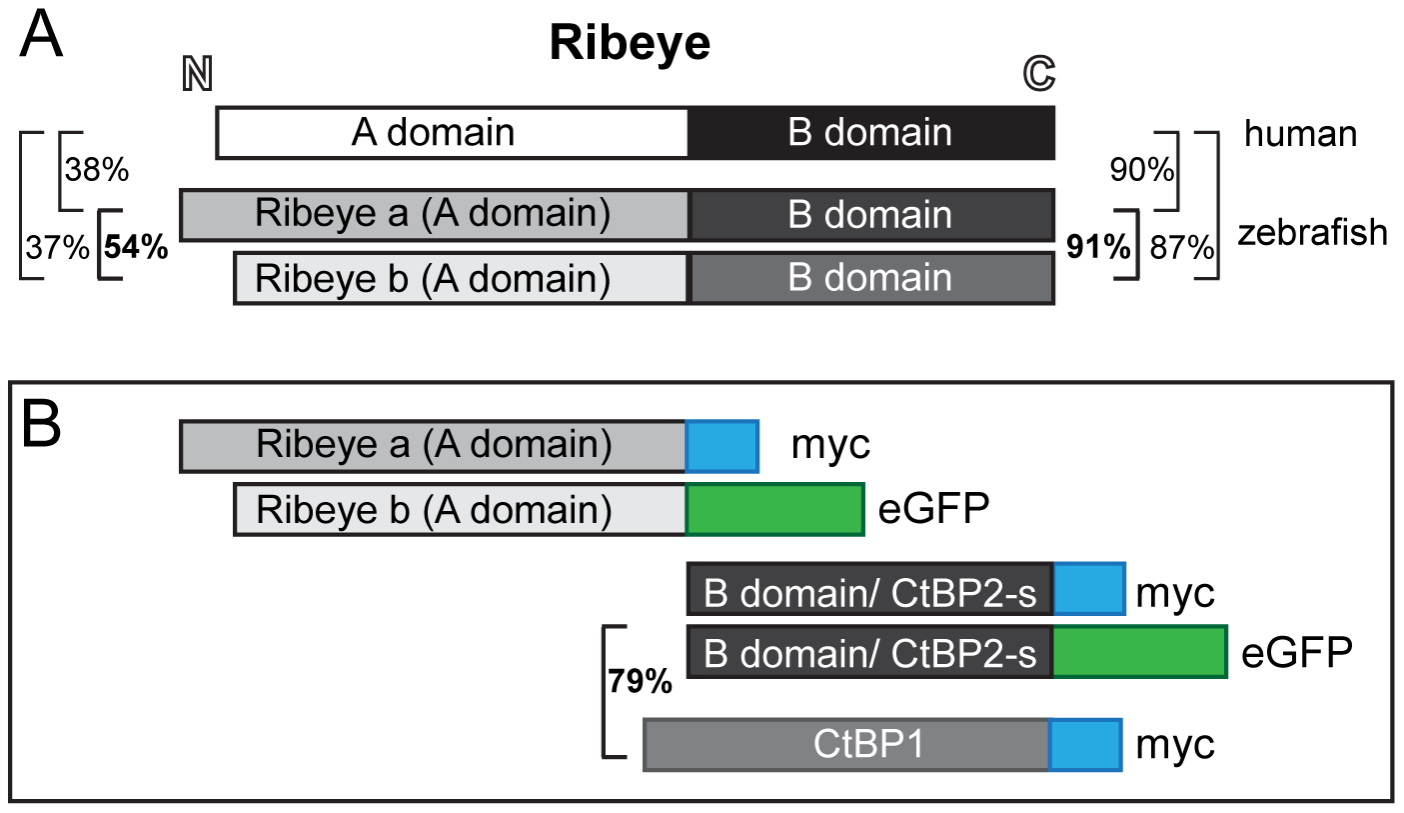
Design of Ribeye A-domain, B-domain/CtBP2s, and CtBP1 constructs. (A) Schematic representation of human and zebrafish Ribeye proteins. Sequence similarity of the two Ribeye domains between human and both zebrafish paralogs are shown as percentages. Protein alignments were performed using the ClustalW method. (B) Schematic representation of the five constructs expressed in the stable transgenic zebrafish lines used in this study. The sequence similarity between Ribeye a B-domain and CtBP1 proteins is shown as a percentage. Hair-cell specific expression of each construct was driven by the *myo6b* promoter [11].

**Figure 2:**
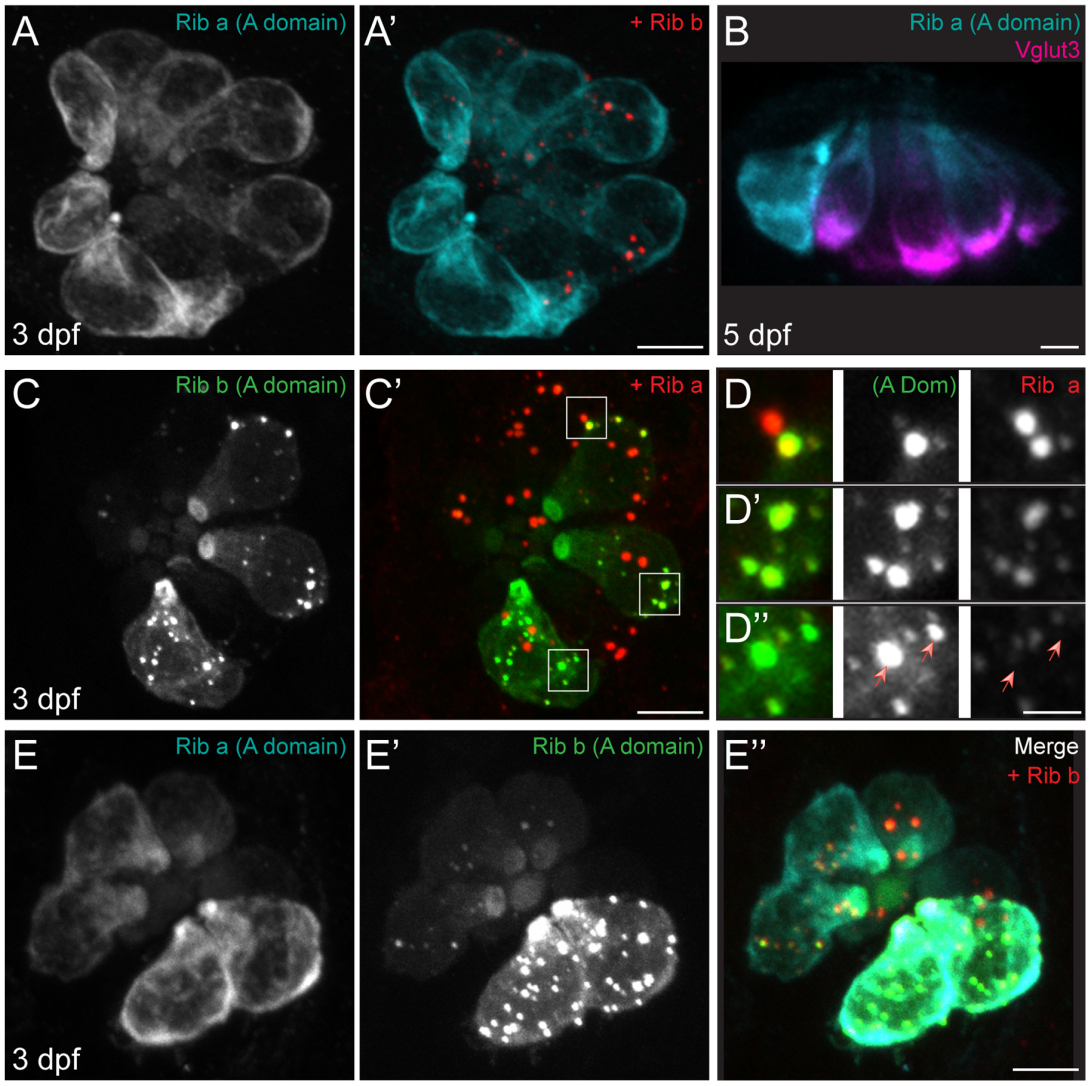
Ribeye A-domains inconsistently localize to synaptic ribbons at 3 dpf and 5 dpf. Representative confocal maximum-intensity projection images of immunolabel or GFP in lateral line NM hair cells. Scale bars: 3 µm (main panels), 1 µm (D, inset). (A–A’) Ribeye a (A-domain)-myc alone (A) and merged with Ribeye b labeling synaptic ribbons (A’) in posterior lateral line NM1 hair cells of a 3 dpf larva. (B) Ribeye a (A-domain)-myc (cyan) and Vglut3 (magenta) immunolabel in a cross-section of an anterior lateral line NM at 5 dpf. (C–C’) Ribeye b (A-domain)-GFP alone (C) and merged with Ribeye a immunolabel (C’) in NM1 hair cells of a 3 dpf larva. White boxes indicate regions used in (D–D”). (D–D”) Insets of region in C’ showing basally-localized Ribeye b (A-domain)-GFP in relation to synaptic ribbons labeled with an antibody to Ribeye a. (D) Two adjacent hair cells: one with low Ribeye b (A-domain) expression next to another hair cell with moderate expression. A domain-GFP colocalizes with endogenous Ribeye a. (D’) A hair cell with moderate Ribeye b (A-domain) expression. (A domain)-GFP colocalizes with endogenous Ribeye a. (D”) A hair cell with high Ribeye b (A-domain) expression. Read arrows indicate basally localized A-domain aggregates that do not colocalize with endogenous Ribeye. (E–E”) Ribeye a (A-domain)-myc (E), Ribeye b (A-domain)-GFP (E’), and merged with Ribeye b immunolabel (E”) in NM1 hair cells of a 3 dpf larva. Note that the Ribeye b antibody labels both endogenous Ribeye b and exogenous Ribeye b (A-domain).

Zebrafish have two copies of the gene encoding Ribeye: *ribeye a and ribeye b* [16], and both Ribeye paralogs are found in zebrafish hair-cell synaptic ribbons [5]. As the sequence similarity between the A-domains of the two Ribeye proteins is relatively low (Figure 1A; 54%), we created constructs containing the A-domain of each Ribeye paralog: *ribeye a (A-domain)* fused to a c-terminal 6x-myc tag and *ribeye b (A-domain)* fused to a c-terminal GFP tag (Figure 1B). Expression of these constructs was driven by the hair-cell specific *myosin6b* promoter [11]. In contrast to what was observed when the A-domain was heterologously expressed in cell lines, exogenously expressed Ribeye a (A-domain)-myc in hair cells did not self-associate nor did it localize to synaptic ribbons (Figure 2A, A’, B). Instead, it localized diffusely throughout the cell bodies of both relatively immature (Figure 2A,A’) and mature (Figure 2B) hair cells. In addition, Ribeye a (A-domain)-myc did not colocalize with the synaptic vesicle marker Vglut3 (Figure 2B), which supports that exogenous Ribeye a (A-domain) failed to traffic to synaptic ribbons. By contrast, Ribeye b (A-domain)-GFP localization in hair cells was variable; it was able to partially self-associate (Figure 2C) and, when expression levels were moderate, localize to synaptic ribbons (Figure 2C’, D, D’). However, we also observed Ribeye b (A-domain)-GFP aggregates that did not localize to synaptic ribbons (Figure 2D”). These results reveal a disparity between the two zebrafish paralogs of A-domain with regards to their self-association and trafficking to synaptic ribbons in zebrafish hair cells.

Because duplicate genes in zebrafish often recapitulate the functions of a single ancestral gene [25], we examined whether co-expression of both Ribeye (A-domain) paralogs would improve their localization to synaptic ribbons. To test this, we crossed transgenic carriers of both *ribeye a (A-domain)-6xmyc* and *ribeye b (A-domain)-gfp*, then examined their localization to synaptic ribbons. Because both of the antibodies we use to label synaptic ribbons interact with the A-domains of each paralog of Ribeye [5], we were unable to label synaptic ribbons without also labeling one of the transgenes. Since labeling Ribeye a (A domain) appeared as a strong cell fill and obscured intracellular structures, we used the antibody against Ribeye b to label synaptic ribbons. In hair cells also expressing Ribeye b (A-domain)-GFP we still observed no synaptic localization of Ribeye a (A-domain)-myc (Figure 2E, E”). Furthermore, we did not observe any apparent improvement in the synaptic localization of Ribeye b (A domain)-GFP when co-expressed with the other A-domain paralog; that is, it appeared to localize to ribbon synapses to a certain extent, but also was found diffusely throughout the hair cells and accumulated at the apical end of hair cells when expressed alone or with Ribeye a (A-domain)-myc (Figure 2C, E’). These results suggest that the Ribeye (A-domain) alone is not completely sufficient to localize to synaptic ribbons.

### Ribeye B-Domain/CtBP2s localizes to synaptic ribbons in developing zebrafish hair cells

**Figure 3:**
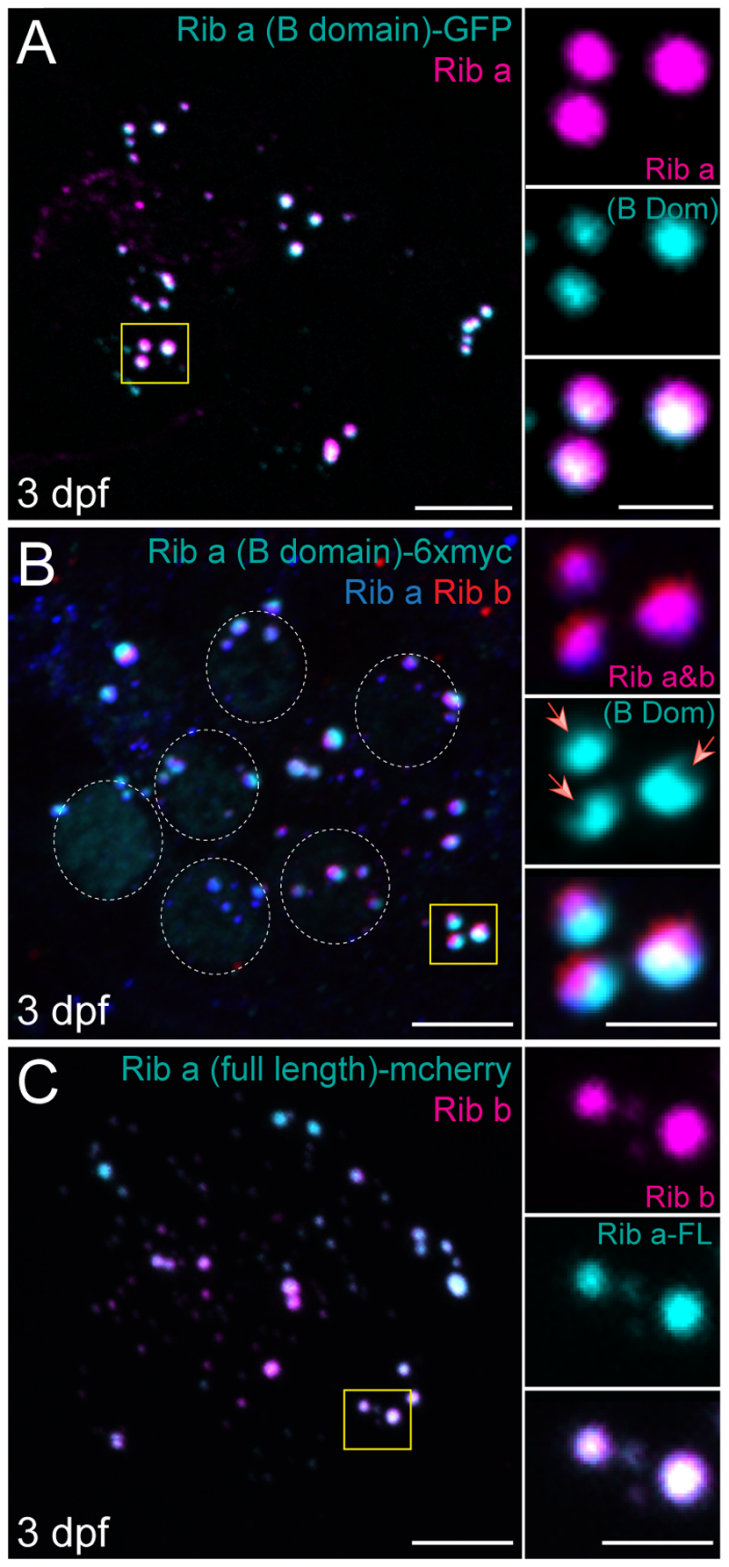
Ribeye B-domain/CtBP2s localizes to synaptic ribbons at 3 dpf. Representative images of immunolabel or fluorescent tag in posterior lateral line NM1 hair cells of 3 dpf larvae. Scale bars: 3 µm (main panels), 1 µm (insets). (A) Ribeye (B-domain)-GFP (cyan) and Ribeye a antibody labeling of synaptic ribbons (magenta). Note that B-domain-GFP does not completely colocalize with Ribeye a immunolabel. (B) Ribeye (B-domain)-myc (cyan), Ribeye a (blue), and Ribeye b (red) immunolabel. Dashed circles indicate nuclear localization of B-domain. Note that Ribeye a and b immunolabel colocalizes with each other (magenta), but only partially colocalizes with B-domain (white). Red arrows indicate resolvable indentations in the synaptic ribbons that do not contain exogenous B-Domain, but do contain Ribeye. (C) Ribeye a (full length)-mcherry (cyan) and Ribeye b immunolabel (magenta). Full length exogenous Ribeye a-mcherry appears throughout the synaptic ribbon and colocalizes with Ribeye b immunolabel (white).

We next examined the localization of Ribeye (B-domain)/CtBP2s by creating transgenic zebrafish lines expressing *ribeye a (B-domain)* fused to either 6x-myc or GFP in hair cells (Figure 1B). Because the sequence similarity between the two zebrafish B-domain/CtBP2s paralogs is high (Figure 1A; 91%), we only created constructs containing the B-domain of one Ribeye paralog. As with the A-domain constructs, expression of B-domain containing constructs was driven by the *myosin6b* promoter. In developing 3-day-old NM hair cells, we observed robust localization of B-domain/CtBP2s to synaptic ribbons within all hair cells examined in both transgenic lines (Figure 3A, B). We also observed weak localization of B-domain/CtBP2s-myc to hair-cell nuclei (Figure 3B; outlined with dashed circles), which is similar to what has been reported in CtBP2s-flag expressing HeLa and COS cells [20]. Strikingly, the B-domain appeared to preferentially localize to one side of ribbon bodies; ribbon-localized B-domain puncta appeared juxtaposed to endogenous Ribeye-containing puncta, but did not fully co-localize (Figure 3A, B; insets). To address whether the apparent substructural localization of the B-domain was an artifact of protein overexpression or the C-terminal tag, we exogenously expressed full-length Ribeye a fused to mCherry and saw complete co-localization of Ribeye a-mCherry and endogenous Ribeye b immunolabel (Figure 3C). We also previously observed comparable results with stably expressed Ribeye b-GFP [5]. Collectively, these results demonstrate that Ribeye (B-domain)/CtBP2s alone can localize to synaptic ribbons and reveal that B-domain localizes to one side of synaptic ribbons. The observation that exogenous B-domain was not distributed throughout synaptic ribbons in a similar way to exogenous full-length Ribeye is noteworthy because it suggests that B-domain alone interacts with synaptic-ribbon components in a different way than full-length Ribeye.

### Ribeye B-domain/CtBP2s disrupts endogenous Ribeye retention in the synaptic ribbons of relatively mature zebrafish hair cells

**Figure 4:**
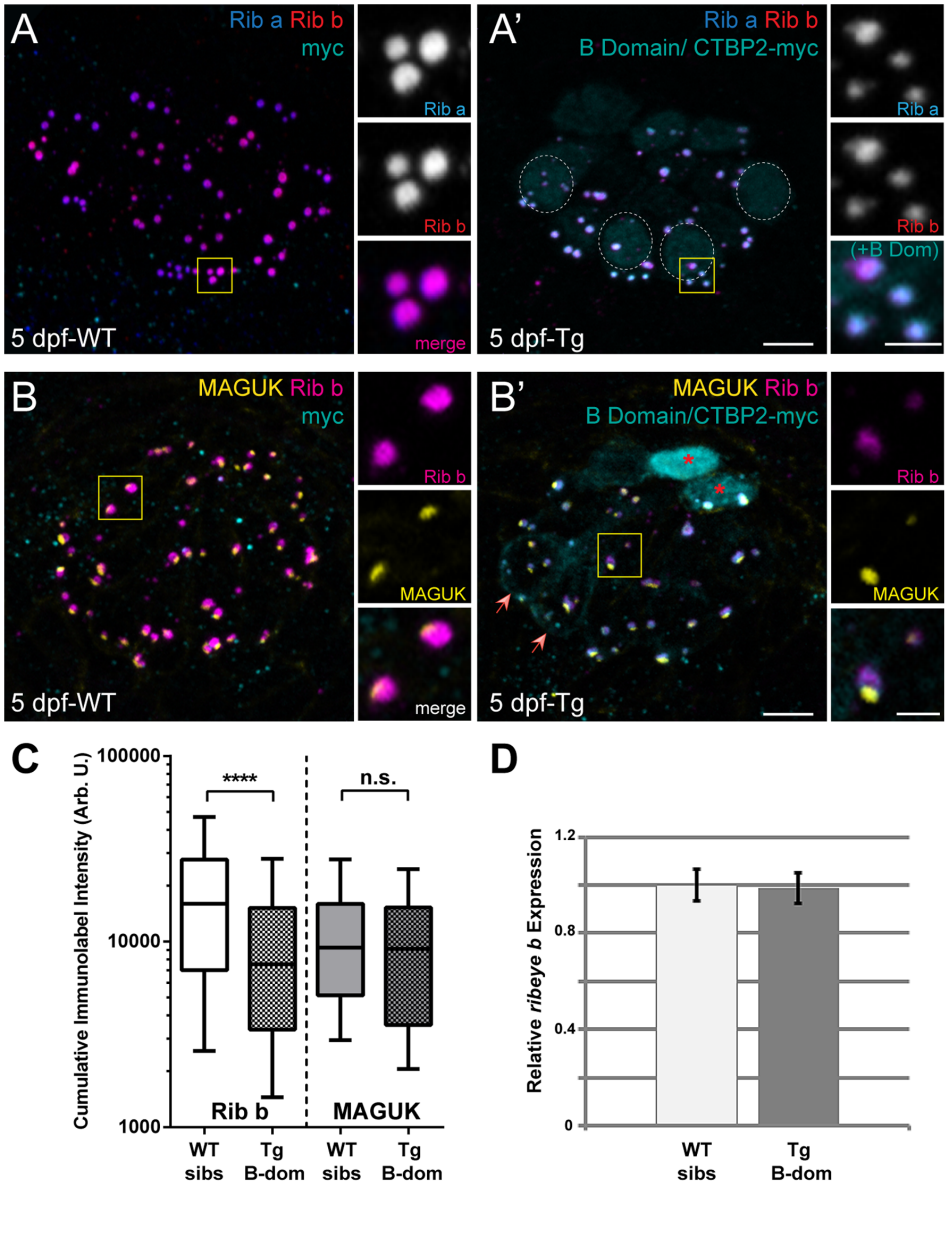
Ribeye B-domain/CtBP2s disrupts endogenous Ribeye retention at synaptic ribbons in 5 dpf hair cells. (A–B) Representative images of immunolabel or fluorescent tag in posterior lateral line NM3 hair cells of 5 dpf larvae. Scale bars: 3 µm (main panels), 1 µm (insets). (A, A’) Ribeye a (blue) and Ribeye b (red) antibody labeling of synaptic ribbons, and anti-myc antibody labeling of B domain-myc (cyan) in a WT (A) and a transgenic (A’) larva. Dashed circles indicate weak nuclear localization of B-domain. (B, B’) Ribeye (B-domain)-myc (cyan), Ribeye b (magenta), and MAGUK (yellow) immunolabel in a WT (B) and a transgenic (B’) larva. Red asterisks indicate strong nuclear localization of B-domain. Red arrows indicate cells with moderate levels of B-domain in the cytosol. (C) Box plots of cumulative immunolabel intensities of presynaptic Ribeye immunolabeled spheres and postsynaptic MAGUK immunolabeled patches in 5 dpf transgenic B domain-myc larvae and WT siblings. These plots show the median value (horizontal bar), the upper and lower quartiles (box), and the range (whiskers). Whiskers indicate the 10^th^ and 90^th^ percentiles. ****P<0.0001, defined by a Mann-Whitney U Test. Each plot represents a population of intensity measurements collected from NM3 hair cells of 7–8 individual larvae. (D) Relative expression level of *ribeye b* transcripts in the posterior lateral line of 5 dpf transgenic B domain-myc larvae and WT siblings. Expression data was normalized to *b-actin* expression. The level of gene expression in WT siblings was normalized to one. Error bars are s.e.m.

To determine whether the pattern of B-domain localization in hair cells persisted at later stages, we examined the localization of Ribeye B-domain/CtBP2s in the relatively more mature hair cells of 5 dpf zebrafish larvae [24], [26]. Similar to what we observed in 3 dpf larvae, both B-domain/CtBP2s transgenes localized well to synaptic ribbons (Figure 4A’, B’). In addition, B-domain/CtBP2s-myc sometimes localized to hair-cell nuclei (17 out of 107 hair cells), but in general was excluded from the nucleus in 5-day-old NM hair cells (Figure 4A’, B’). Notably, the immunolabel intensity of both endogenous Ribeye a and b at presumptive presynaptic ribbons appeared reduced in B-domain/CtBP2s expressing hair cells compared to WT siblings at 5 dpf (Figure 4A, A’), which we did not observe at 3 dpf (Figure 3 A, B). To quantify the degree of reduction in ribbon-synapse localized Ribeye, we used Amira image processing software to create 3D-renderings of confocal stacks. We then measured the cumulative intensity of each Ribeye immunolabeled sphere adjacent to a patch of postsynaptic density labeled with an antibody against the PSD-95 family of membrane associated guanylate kinases (MAGUKs) (Figure 4 B, B’). We observed a significant reduction in the cumulative pixel intensity of endogenous Ribeye at presynaptic spheres (Figure 4C; Mann Whitney U test: P<0.0001) but not MAGUK at postsynaptic patches (Mann Whitney U test: P = 0.2504). This indicates that exogenous B-domain reduces endogenous Ribeye protein levels within synaptic ribbons but does not disrupt postsynaptic components. Because we observed nuclear localization of exogenous B-domain/CtBP2s and CtBP2 is a transcriptional corepressor, we sought to address whether B-domain overexpression reduced transcript levels of *ribeye* in 5-day-old zebrafish hair cells. We therefore performed qPCR and saw no difference in the relative amount of *ribeye b* transcript in B-domain overexpressing larvae versus wild-type (Figure 4D), supporting the notion that exogenous B-domain/CtBP2s disrupts retention of Ribeye at synaptic ribbons.

**Figure 5:**
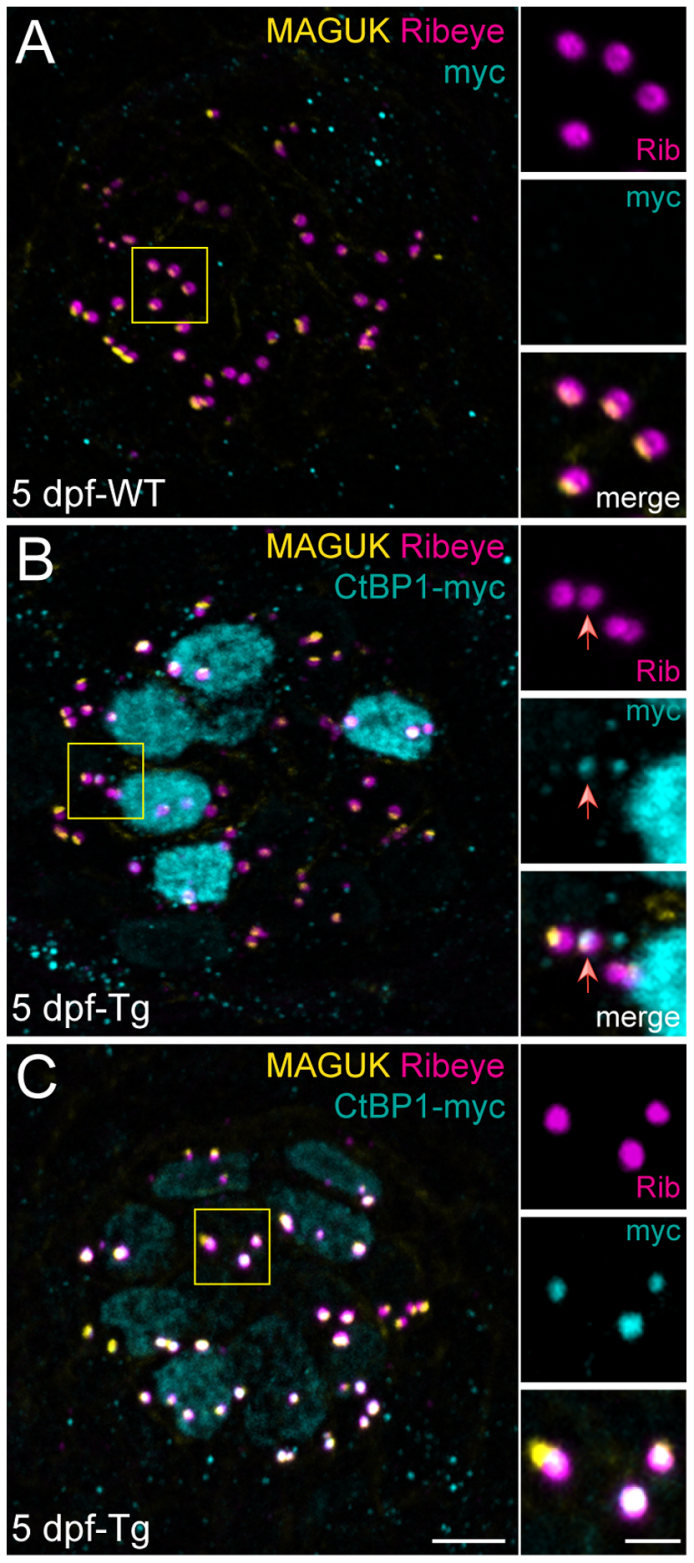
CtBP1 localizes to synaptic ribbons, but does not disrupt endogenous Ribeye. Representative images of immunolabel in posterior lateral line NM3 hair cells of 5 dpf larvae. Scale bars: 3 µm (main panels), 1 µm (insets). (A) Ribeye b antibody labeling of synaptic ribbons (magenta) and MAGUK antibody labeling of postsynaptic densities (yellow) in a WT sibling larva. Anti-myc (cyan) immunolabel was performed as a negative control. (B–C) CtBP1-myc (cyan), Ribeye b (magenta), and MAGUK (yellow) immunolabel in two representative transgenic larvae. (B) CtBP1-myc (cyan) is strongly localized to the nucleus with weak synaptic localization. The red arrow indicates a synaptic ribbon containing CtBP1-myc. Note that Ribeye immunolabel intensity in all four synaptic ribbons appears comparable. (C) CtBP1-myc (cyan) is weakly localized to the nucleus with strong synaptic localization. Presynaptic Ribeye immunolabel intensity is not reduced compared to WT (A).

CtBP2 shares a high degree of sequence similarity with the related transcriptional corepressor CtBP1 (Figure 1B; 79%). CtBP1 has been shown to heterodimerize with CtBP2 [20] and is also a component of synaptic ribbons [27], [28], [29], [30]. Therefore, we tested whether exogenously expressed CtBP1 would localize to synaptic ribbons in a similar way as B-domain/CtBP2s in zebrafish hair cells. We observed that exogenous CtBP1 also localized to hair-cell nuclei and to synaptic ribbons (two examples shown in Figure 5B, C). In contrast to what we observed with B-domain/CtBP2s (Figure 4B’; inset), exogenous expression of CtBP1 did not reduce endogenous Ribeye immunolabel at synaptic ribbons (Figure 5B, C; insets). These data suggest that the reduction of Ribeye at synaptic ribbons we observed in hair cells expressing Ribeye B-domain/CtBP2s is specific to CtBP2s and not just a consequence of excessive CtBP at the synaptic ribbon.

### B-domain/CtBP2s and CtBP1 localize to the basal end of synaptic ribbon bodies adjacent to postsynaptic densities

**Figure 6:**
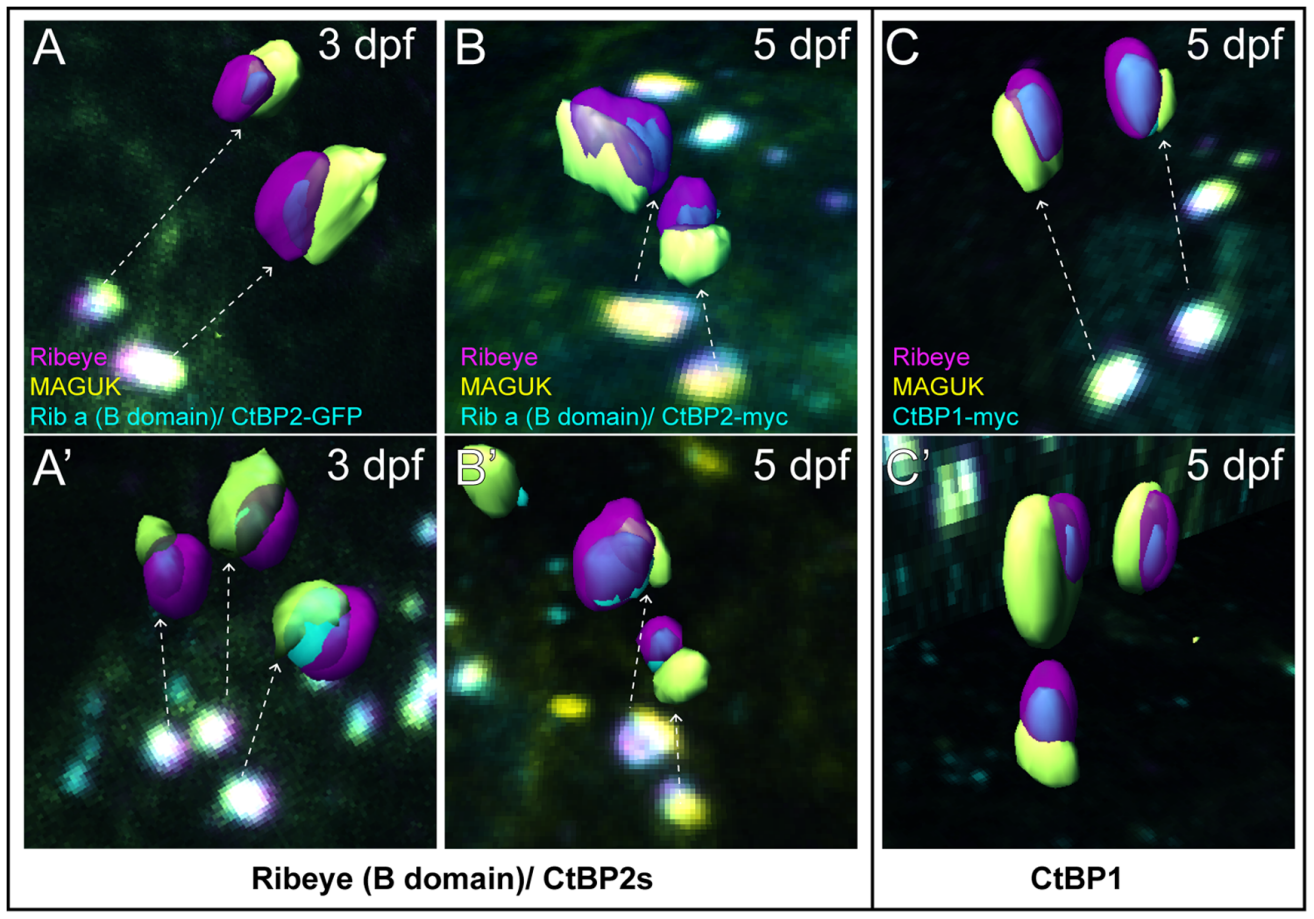
Ribeye B-domain/CtBP2s and CtBP1 localizes to the basal end of synaptic ribbons facing the postsynaptic density. Isosurface renderings of ribbon synapses extrapolated from z-stack confocal images of Ribeye b (magenta), GFP or myc (cyan), and MAGUK (yellow). Dashed arrows indicate the ribbon synapses used from the images to generate the 3D renderings. (A–A’) Ribeye (B-domain)-GFP (cyan) with Ribeye b (magenta), and MAGUK (yellow) in 3 dpf larvae. Note that B-domain-GFP within synaptic ribbons appears adjacent to patches of MAGUK. (B–B’) Ribeye (B-domain)/CtBP2s-myc (cyan) with Ribeye b (magenta), and MAGUK (yellow) in 5 dpf larvae. Note that B-domain-myc within synaptic ribbons also appears adjacent to patches of MAGUK. (C–C’) CtBP1-myc (cyan) with Ribeye b (magenta), and MAGUK (yellow) in 5 dpf larvae. Synaptic ribbon localization of CtBP1 appears comparable to Ribeye (B-domain)/CtBP2s.

Our experiments indicated that B-domain/CtBP2s is enriched on one side of synaptic ribbons in 3 dpf larvae (Figure 3 A, B). To visualize the localization of the B-domain/CtBP2s with respect to the postsynaptic density, we generated 3-D isosurface renderings of ribbon synapses in the hair cells of both 3- and 5-day-old transgenic larvae. While full-length endogenous Ribeye was generally found throughout the synaptic ribbon, B-domain/CtBP2s-GFP and B-domain/CtBP2s-myc were localized to a section of the ribbon body adjacent to the postsynaptic density at both larval ages examined (Figure 6A, B). Moreover, CtBP1-myc also localized to an area of synaptic ribbons adjacent to postsynaptic densities (Figure 6C), suggesting that, in general, endogenous ribbon-localized CtBPs may be preferentially segregated to the basal end of synaptic ribbons in zebrafish hair cells.

## Discussion

Self-association of the protein RIBEYE, which is mediated by homo- and heterotypic interactions between its two domains [17], is critical to synaptic-ribbon formation and plasticity. While regulation of RIBEYE domain interactions appears to play an important role in synaptic ribbon assembly [3], the behavior of individual RIBEYE domains within sensory cells has not been previously described. In the present study, we characterized the localization of exogenously expressed Ribeye A- and B-domains and the closely related protein CtBP1 within lateral line hair cells of stable transgenic zebrafish. We observed that *(i)* Ribeye a (A-domain) fails to self-associate or localize to synapses, *(ii)* Ribeye b (A-domain) incompletely self-associates, but generally localizes to synapses, *(iii)* both B-domain/CtBP2s and CtBP1 localize to the nucleus, the cytosol, and synaptic ribbons, *(iv)* B-domain/CtBP2s disrupts endogenous Ribeye retention at synaptic ribbons in 5-day-old hair cells, and *(v)* CtBP1 and B-domain/CtBP2s localize to the basal end of synaptic ribbons adjacent to the postsynaptic density. Collectively, these results suggest that zebrafish Ribeye A-domains do not contain all the required elements needed to completely localize to synaptic ribbons, while Ribeye B-domain contains elements that enable effective localization to synaptic ribbons such that it may displace endogenous Ribeye in mature zebrafish hair cells.

The incomplete self-assembly and variable association of Ribeye A-domains with synaptic ribbons suggest that multiple cellular mechanisms regulate A-domain association with itself and/or full-length Ribeye *in vivo*, which is in contrast to the robust and complete self-assembly of RIBEYE A-domain observed in cell culture. However, the two zebrafish paralogs of A-domain share only ∼50% sequence similarity both with each other and with human RIBEYE A-domain (Figure 1A), which may explain why they differ greatly in their self-association and localization to synaptic ribbons As both zebrafish paralogs of Ribeye are distributed throughout hair-cell synaptic ribbons and the assembly of each paralog is comparably regulated during hair-cell ribbon maturation [24], it is conceivable that an additional level of cellular regulation exists in zebrafish to coordinate the assembly of both paralogs of Ribeye that may differ from the regulation of mammalian RIBEYE A-domain assembly *in vivo*.

By contrast, zebrafish Ribeye B-domain, which shares a high degree of sequence similarity with mammalian B-domain, appears to associate with synaptic ribbon structures in hair cells in a similar way as previously described in heterologous expression systems; that is, it associates with full-length Ribeye [17]. There are, however, a couple of notable and interesting differences. One difference is that ribbon-localized B-domain did not distribute evenly throughout synaptic ribbons and did not completely overlap with full-length Ribeye. Rather, B-domain accumulated to the basal side of synaptic ribbons facing the post-synaptic density (Figure 6A, B). Another difference observed was that as hair cell matured, the B-domain appeared to disrupt endogenous Ribeye accumulation at synaptic ribbons (Figure 4A–C). Cumulatively, these data suggest additional cellular mechanisms regulating molecular interactions of the B-domain with other components of the synaptic ribbons.

The observation that exogenous B-domain/CtBP2s and CtBP1 accumulated within synaptic ribbons adjacent to postsynaptic densities may reflect the behavior of endogenous CtBP proteins in sensory hair cells. Both CtBP1 and CtBP2s have been found to localize to conventional presynapses throughout the brain [27], [31], supporting that CtBPs may be generally conserved active zone molecules. With regards to ribbon synapses, CtBP1 is also a component of synaptic ribbons, and both CtBP1 and B-domain/CtBP2s directly interact with each other and with Bassoon–an active-zone protein that is required for maintenance of synaptic-ribbon attachment to the plasma membrane [27], [32], [33]. Speculatively, enrichment of exogenous B-domain/CtBP2s and CtBP1 at the base of hair-cell synaptic ribbons may reflect their interaction with Bassoon near the plasma membrane. It is important to note that, in contrast to what we observed in hair-cell synaptic ribbons, a previous study examining synaptic ribbon components in photoreceptors reported enrichment of Bassoon immunogold labeling at the base of synaptic ribbons, yet CtBP1 immunogold labeling was present throughout photoreceptor synaptic ribbons in EM micrographs [27]. Further studies examining the localization of endogenous CtBP1 in hair cells will clarify whether the substructural synaptic localization of exogenous CtBP1 that we observed truly reflects its native localization in hair-cell synapses.

While CtBP1is indisputably part of the synaptic ribbon protein complex, there exist conflicting reports as to whether CtBP2 is also a component of synaptic ribbons [28], [29], [30]. As sensory hair cells certainly contain CtBP2 [6], [34] and considering that B-domain/CtBP2s appears to disrupt native Ribeye localization at ribbon synapses, there are likely regulatory mechanisms within hair cells that limit or exclude CtBP2 from synaptic ribbons. It may be that our exogenously expressed B-domain/CtBP2s overrode these presumptive regulatory mechanisms either by overabundance of the protein or because the B-domain/CtBP2s transgene we used lacks the N-terminal nuclear localization signal, which has been reported as the main determinant of sorting CtBP2 to the nucleus instead of the presynaptic active zone [31]. Interestingly, the notion that B-domain/CtBP2s outcompetes native Ribeye is supported by evidence that physiological levels of NAD(H) negatively regulate heterotypic interactions between Ribeye A-domain and B-domain [17], thereby favoring homodimerization between the B-domains. Because synaptic-ribbon size is tightly regulated in zebrafish hair cells [24], we speculate that B-domain/CtBP2s may occupy a number of the limited slots for Ribeye via homotyptic interactions between full-length endogenous Ribeye’s B-domain and exogenous B-domain.

In conclusion, our results suggest that multiple mechanisms exist in sensory cells that regulate both Ribeye self-association and Ribeye’s interaction with CtBP1 at synaptic ribbons. Our results also raise the possibility that the organization of CtBP proteins within synaptic ribbons may be compartmentalized. It is important to note that overexpression of Ribeye subunits or CtBP1 did not grossly affect 5-day-old zebrafish hearing or balance, however we did not test for subtle defects in auditory or vestibular function. Future studies addressing whether and how an overabundance of CtBP1 or CtBP2 affect ribbon-synapse activity could give important insight into the functional organization of synaptic ribbons.

## References

[1] Fuchs PA, Glowatzki E, Moser T (2003) The afferent synapse of cochlear hair cells. Current opinion in neurobiology 13: 452–458. 12965293 10.1016/s0959-4388(03)00098-9

[2] Kim MH, Li GL, von Gersdorff H (2013) Single Ca2+ channels and exocytosis at sensory synapses. The Journal of physiology 591: 3167–3178. 23459757 10.1113/jphysiol.2012.249482 PMC3717220

[3] Schmitz F (2009) The making of synaptic ribbons: how they are built and what they do. The Neuroscientist: a review journal bringing neurobiology, neurology and psychiatry 15: 611–624. 10.1177/1073858409340253 19700740

[4] Safieddine S, El-Amraoui A, Petit C (2012) The auditory hair cell ribbon synapse: from assembly to function. Annual review of neuroscience 35: 509–528. 10.1146/annurev-neuro-061010-113705 22715884

[5] Sheets L, Trapani JG, Mo W, Obholzer N, Nicolson T (2011) Ribeye is required for presynaptic Ca(V)1.3a channel localization and afferent innervation of sensory hair cells. Development 138: 1309–1319. 21350006 10.1242/dev.059451 PMC3050663

[6] Frank T, Rutherford MA, Strenzke N, Neef A, Pangrsic T, et al (2010) Bassoon and the synaptic ribbon organize Ca(2)+ channels and vesicles to add release sites and promote refilling. Neuron 68: 724–738. 21092861 10.1016/j.neuron.2010.10.027 PMC3005353

[7] Wittig JH Jr, Parsons TD (2008) Synaptic ribbon enables temporal precision of hair cell afferent synapse by increasing the number of readily releasable vesicles: a modeling study. Journal of neurophysiology 100: 1724–1739. 18667546 10.1152/jn.90322.2008 PMC2576205

[8] Snellman J, Mehta B, Babai N, Bartoletti TM, Akmentin W, et al (2011) Acute destruction of the synaptic ribbon reveals a role for the ribbon in vesicle priming. Nature neuroscience 14: 1135–1141. 21785435 10.1038/nn.2870 PMC3171202

[9] Schnee ME, Santos-Sacchi J, Castellano-Munoz M, Kong JH, Ricci AJ (2011) Calcium-dependent synaptic vesicle trafficking underlies indefatigable release at the hair cell afferent fiber synapse. Neuron 70: 326–338. 21521617 10.1016/j.neuron.2011.01.031 PMC3254016

[10] Nouvian R, Beutner D, Parsons TD, Moser T (2006) Structure and function of the hair cell ribbon synapse. The Journal of membrane biology 209: 153–165. 16773499 10.1007/s00232-005-0854-4 PMC1764598

[11] Obholzer N, Wolfson S, Trapani JG, Mo W, Nechiporuk A, et al (2008) Vesicular glutamate transporter 3 is required for synaptic transmission in zebrafish hair cells. The Journal of neuroscience: the official journal of the Society for Neuroscience 28: 2110–2118. 18305245 10.1523/JNEUROSCI.5230-07.2008 PMC6671858

[12] Vollrath L, Spiwoks-Becker I (1996) Plasticity of retinal ribbon synapses. Microscopy research and technique 35: 472–487. 9016450 10.1002/(SICI)1097-0029(19961215)35:6<472::AID-JEMT6>3.0.CO;2-K

[13] Spiwoks-Becker I, Glas M, Lasarzik I, Vollrath L (2004) Mouse photoreceptor synaptic ribbons lose and regain material in response to illumination changes. The European journal of neuroscience 19: 1559–1571. 15066152 10.1111/j.1460-9568.2004.03198.x

[14] Spiwoks-Becker I, Maus C, tom Dieck S, Fejtova A, Engel L, et al (2008) Active zone proteins are dynamically associated with synaptic ribbons in rat pinealocytes. Cell and tissue research 333: 185–195. 18523806 10.1007/s00441-008-0627-3 PMC2757586

[15] Hull C, Studholme K, Yazulla S, von Gersdorff H (2006) Diurnal changes in exocytosis and the number of synaptic ribbons at active zones of an ON-type bipolar cell terminal. Journal of neurophysiology 96: 2025–2033. 16738212 10.1152/jn.00364.2006 PMC3572854

[16] Wan L, Almers W, Chen W (2005) Two ribeye genes in teleosts: the role of Ribeye in ribbon formation and bipolar cell development. The Journal of neuroscience: the official journal of the Society for Neuroscience 25: 941–949. 15673675 10.1523/JNEUROSCI.4657-04.2005 PMC6725632

[17] Magupalli VG, Schwarz K, Alpadi K, Natarajan S, Seigel GM, et al (2008) Multiple RIBEYE-RIBEYE interactions create a dynamic scaffold for the formation of synaptic ribbons. The Journal of neuroscience: the official journal of the Society for Neuroscience 28: 7954–7967. 18685021 10.1523/JNEUROSCI.1964-08.2008 PMC6670776

[18] Schmitz F, Konigstorfer A, Sudhof TC (2000) RIBEYE, a component of synaptic ribbons: a protein’s journey through evolution provides insight into synaptic ribbon function. Neuron 28: 857–872. 11163272 10.1016/s0896-6273(00)00159-8

[19] Zenisek D, Horst NK, Merrifield C, Sterling P, Matthews G (2004) Visualizing synaptic ribbons in the living cell. The Journal of neuroscience: the official journal of the Society for Neuroscience 24: 9752–9759. 15525760 10.1523/JNEUROSCI.2886-04.2004 PMC6730242

[20] Verger A, Quinlan KG, Crofts LA, Spano S, Corda D, et al (2006) Mechanisms directing the nuclear localization of the CtBP family proteins. Molecular and cellular biology 26: 4882–4894. 16782877 10.1128/MCB.02402-05 PMC1489157

[21] Regus-Leidig H, Specht D, Tom Dieck S, Brandstatter JH (2010) Stability of active zone components at the photoreceptor ribbon complex. Molecular vision 16: 2690–2700. 21179232 PMC3002953

[22] Westerfield M (1993) The zebrafish book: a guide for the laboratory use of zebrafish (Brachydanio rerio). Eugene, OR: M. Westerfield.

[23] Kwan KM, Fujimoto E, Grabher C, Mangum BD, Hardy ME, et al (2007) The Tol2kit: a multisite gateway-based construction kit for Tol2 transposon transgenesis constructs. Developmental dynamics: an official publication of the American Association of Anatomists 236: 3088–3099. 17937395 10.1002/dvdy.21343

[24] Sheets L, Kindt KS, Nicolson T (2012) Presynaptic CaV1.3 channels regulate synaptic ribbon size and are required for synaptic maintenance in sensory hair cells. The Journal of neuroscience: the official journal of the Society for Neuroscience 32: 17273–17286. 23197719 10.1523/JNEUROSCI.3005-12.2012 PMC3718275

[25] Prince VE, Pickett FB (2002) Splitting pairs: the diverging fates of duplicated genes. Nature reviews Genetics 3: 827–837. 10.1038/nrg928 12415313

[26] Santos F, MacDonald G, Rubel EW, Raible DW (2006) Lateral line hair cell maturation is a determinant of aminoglycoside susceptibility in zebrafish (Danio rerio). Hearing research 213: 25–33. 16459035 10.1016/j.heares.2005.12.009

[27] tom Dieck S, Altrock WD, Kessels MM, Qualmann B, Regus H, et al (2005) Molecular dissection of the photoreceptor ribbon synapse: physical interaction of Bassoon and RIBEYE is essential for the assembly of the ribbon complex. The Journal of cell biology 168: 825–836. 15728193 10.1083/jcb.200408157 PMC2171818

[28] Uthaiah RC, Hudspeth AJ (2010) Molecular anatomy of the hair cell’s ribbon synapse. The Journal of neuroscience: the official journal of the Society for Neuroscience 30: 12387–12399. 20844134 10.1523/JNEUROSCI.1014-10.2010 PMC2945476

[29] Kantardzhieva A, Peppi M, Lane WS, Sewell WF (2012) Protein composition of immunoprecipitated synaptic ribbons. Journal of proteome research 11: 1163–1174. 22103298 10.1021/pr2008972 PMC3274173

[30] Schwarz K, Natarajan S, Kassas N, Vitale N, Schmitz F (2011) The synaptic ribbon is a site of phosphatidic acid generation in ribbon synapses. The Journal of neuroscience: the official journal of the Society for Neuroscience 31: 15996–16011. 22049442 10.1523/JNEUROSCI.2965-11.2011 PMC6623010

[31] Hubler D, Rankovic M, Richter K, Lazarevic V, Altrock WD, et al (2012) Differential spatial expression and subcellular localization of CtBP family members in rodent brain. PloS one 7: e39710. 22745816 10.1371/journal.pone.0039710 PMC3382178

[32] Dick O, tom Dieck S, Altrock WD, Ammermuller J, Weiler R, et al (2003) The presynaptic active zone protein bassoon is essential for photoreceptor ribbon synapse formation in the retina. Neuron 37: 775–786. 12628168 10.1016/s0896-6273(03)00086-2

[33] Khimich D, Nouvian R, Pujol R, Tom Dieck S, Egner A, et al (2005) Hair cell synaptic ribbons are essential for synchronous auditory signalling. Nature 434: 889–894. 15829963 10.1038/nature03418

[34] Liberman LD, Wang H, Liberman MC (2011) Opposing gradients of ribbon size and AMPA receptor expression underlie sensitivity differences among cochlear-nerve/hair-cell synapses. The Journal of neuroscience: the official journal of the Society for Neuroscience 31: 801–808. 21248103 10.1523/JNEUROSCI.3389-10.2011 PMC3290333

